# Supplementary material for: MetaRibo-Seq measures translation in microbiomes
Source: Nat Commun. 2020 Jun 29;11:3268. doi: 10.1038/s41467-020-17081-z (PMC7324362; doi:10.1038/s41467-020-17081-z)
Supplement: Supplementary file 10 — Supplementary Data 7 [file 41467_2020_17081_MOESM10_ESM.zip › File2/Confidence_High_Taxonomy/395428_out.krona.html]

Javascript must be enabled to view this page.

members
magnitude
magnitudeUnassigned
count
unassigned
taxon
rank

395428\_out

13

2
superkingdom
13

13
phylum
1239

186801
13
class

order
13
186802

13
family
186803

841
genus
13


SRS012849\_contig\_number\_31395SRS014923\_contig\_number\_28037SRS017433\_contig\_number\_contig-100\_459.110268SRS048164\_contig\_number\_31494SRS050925\_contig\_number\_19814SRS051882\_contig\_number\_9913SRS056519\_contig\_number\_12679SRS063040\_contig\_number\_46247SRS064276\_contig\_number\_40927SRS098881\_contig\_number\_6308SRS143417\_contig\_number\_4468SRS148784\_contig\_number\_contig-100\_1869.158397SRS893288\_contig\_number\_1236
species
13
360807
